# Tau exhibits unique seeding properties in globular glial tauopathy

**DOI:** 10.1186/s40478-019-0691-9

**Published:** 2019-03-07

**Authors:** Dah-eun Chloe Chung, Yari Carlomagno, Casey N. Cook, Karen Jansen-West, Lillian Daughrity, Laura J. Lewis-Tuffin, Monica Castanedes-Casey, Michael DeTure, Dennis W. Dickson, Leonard Petrucelli

**Affiliations:** 1 Department of Neuroscience, Mayo Clinic, 4500 San Pablo Road, Jacksonville, FL 32224 USA; 2 Neuroscience Graduate Program, Mayo Clinic Graduate School of Biomedical Sciences, Jacksonville, FL 32224 USA; 3 Department of Cancer Biology, Mayo Clinic, Jacksonville, FL 32224 USA

**Keywords:** Tau, Tauopathy, Globular glial tauopathy, Seeding, Aggregation

## Abstract

Tauopathies are neurodegenerative disorders characterized by aggregation of microtubule associated tau protein in neurons and glia. They are clinically and pathologically heterogeneous depending on the isoform of tau protein that accumulates (three or four 31-to-32-amino-acid repeats [3R or 4R] in the microtubule binding domain), as well as the cellular and neuroanatomical distribution of tau pathology. Growing evidence suggests that distinct tau conformers may contribute to the characteristic features of various tauopathies. Globular glial tauopathy (GGT) is a rare 4R tauopathy with globular cytoplasmic inclusions within neurons and glial cells. Given the unique cellular distribution and morphology of tau pathology in GGT, we sought to determine if tau species in GGT had distinctive biological properties. To address this question, we performed seeding analyses with postmortem brain tissues using a commercial tau biosensor cell line. We found that brain lysates from GGT cases had significantly higher seeding competency than other tauopathies, including corticobasal degeneration (CBD), progressive supranuclear palsy (PSP), and Alzheimer’s disease (AD). The robust seeding activity of GGT brain lysates was independent of phosphorylated tau burden and diminished upon removal of tau from samples, suggesting that seeding properties were indeed mediated by tau in the lysates. In addition, cellular inclusions in the tau biosensor cell line induced by GGT had a distinct, globular morphology that was markedly different from inclusions induced by other tauopathies, further highlighting the unique nature of tau species in GGT. Characterization of different tau species in GGT showed that detergent-insoluble, fibril-like tau contained the highest seeding activity, as reflected in its ability to increase tau aggregation in primary glial cultures. Taken together, our data suggest that unique seeding properties differentiate GGT-tau from other tauopathies, which provides new insight into pathogenic heterogeneity of primary neurodegenerative tauopathies.

**Electronic supplementary material:**

The online version of this article (10.1186/s40478-019-0691-9) contains supplementary material, which is available to authorized users.

## Introduction

Tauopathies are a group of neurodegenerative diseases that are characterized neuropathologically by abnormal deposition of hyperphosphorylated forms of the microtubule-associated tau protein [19, 34]. Although aberrant tau aggregation is an overlapping pathological hallmark, tauopathies exhibit remarkable heterogeneity in both their clinical and neuropathological presentation [19, 34]. For instance, cell types that are susceptible to tau pathology differ among tauopathies as proportion of tau pathology in neurons and glia varies in specific tauopathies, with glial tau pathology being minimal in Alzheimer’s disease (AD), but frequent in primary tauopathies [11, 17]. The presence of different tau isoforms that include either three or four 31- or 32-amino acid repeats in the microtubule binding domain, designated 3R or 4R, further contributes to differences in biochemical properties underlying tau pathology [1, 7, 28, 29]. Indeed, tauopathies are classified based upon the predominant form of abnormal tau in cellular inclusions – 3R, 4R, or 3R + 4R tauopathies [19, 34]. Importantly, multiple studies suggest that distinct tau conformers correlate with differences in tau pathology observed in tauopathies, including the specific brain regions that are affected and characteristic accumulation pattern of tau [5, 18, 21, 27]. As such, the heterogeneous nature of tau pathology in primary and secondary tauopathies complicates our understanding of the exact pathomechanisms that lead to abnormal aggregation and deposition of tau, challenging efforts to develop effective therapeutic interventions.

Globular glial tauopathy (GGT) is a rare 4R tauopathy characterized by globular, tau-positive inclusions in astrocytes and oligodendrocytes called globular glial inclusions (GGIs) [2]. The morphology of GGIs in GGT is distinct from glial abnormalities found in other tauopathies, such as tufted astrocytes in progressive supranuclear palsy (PSP) and astrocytic plaques in corticobasal degeneration (CBD) [19]. GGT cases can be further classified into subtype I, II, or III accompanying different clinical symptoms, based on the distribution pattern of GGIs in the frontal versus motor cortex and/or grey versus white matter, as well as differential astroglial or oligodendroglial involvement in pathological tau inclusions [2]. Due to its rarity, GGT has only recently been defined as a separate disease category [2, 3], and thus tau species in GGT require further study.

Given both the rarity and unique morphology of tau-positive GGIs, we wanted to determine the specific properties that differentiate pathological tau species in GGT from abnormal forms of tau in more common tauopathies, such as AD, PSP, and CBD. In particular, as pathological forms of tau are believed to contribute to the spread of tau pathology by acting as seeds that recruit normal tau and stimulate aggregation [13], in the current study we evaluated the seeding potential of abnormal tau species in GGT. Compared to other tauopathies and healthy controls, brain lysates of GGT demonstrated very strong tau seeding-like activity as monitored by the tau biosensor cell line, in a GGT subtype-specific manner. Through immunodepletion of tau from GGT brain lysates, we confirmed that tau was the main protein component mediating this robust seeding activity, which leads to the formation of tau inclusions with a very distinct morphology reminiscent of GGIs. Moreover, we determined that detergent-insoluble, fibrillar forms of tau isolated from GGT brain samples possess the strongest seeding competency compared to either monomeric or oligomeric GGT-tau. GGT tau filaments also exhibit high seeding competency in primary glial cultures. Taken together, our findings reveal that pathogenic tau species implicated in GGT are characterized by very unique and robust seeding properties that are not detected in other tauopathies, reflecting inherent differences in the tendency of GGT-tau to propagate distinct glial tau pathology.

## Experimental procedures

### Antibodies

We generated E1 antibody for human-specific tau (amino acid residues 19–33 within exon 1 of human tau) [24]. Tau 5 antibody for total mouse and human tau was provided by our late and dear colleague Dr. Skip Binder (Northwestern University Medical School, Chicago, IL). CP13 antibody for phospho-tau (pS202) was kindly provided by Dr. Peter Davies (Feinstein Institute for Medical Research, Northwell Health). We purchased tau monoclonal antibody HT7 from Thermo Fisher (Waltham, MA), anti-GFAP from Cell Signaling Technology, Inc. (Danvers, MA), anti-GFP from Life Technologies (Grand Island, NY), anti-V5 from Invitrogen (Carlsbad, CA), and anti-GAPDH from Meridian Life Science, Inc. (Memphis, TN). Secondary antibodies were purchased from Jackson ImmunoResearch Laboratories, Inc. (West Grove, PA).

### Sample preparation and immunoblotting procedure

Cells were harvested to be lysed in lysis buffer (50 mM Tris HCl [pH 7.4], 274 mM NaCl, 5 mM KCl, 5 mM EDTA, 1% Triton-X-100, 1% SDS, 1 mM PMSF, protease inhibitor cocktail, and phosphatase inhibitor cocktails II and III), followed by sonication. Samples were centrifuged at 16,000 x g for 15 min at 4 °C, and the supernatant was collected for standard BCA protein assay (Pierce Biotechnology, Rockford, IL). Cell lysate (20 μg of protein) was added with 2X Tris-glycine SDS sample buffer (Life Technologies), 5% beta-mercaptoethanol (Sigma-Aldrich, St. Louis, MO), and dH_2_O. After heat-denaturation for 5 min at 95 °C, samples were run on SDS-PAGE Tris-glycine gels (Life Technologies), and transferred to PVDF membrane (Millipore, Burlington, MA). Membranes were blocked in 5% non-fat dry milk in TBS/0.1% Triton-X-100, and incubated with primary antibody rocking overnight at 4 °C. Subsequently, membranes were incubated with HRP-conjugated secondary antibodies (1:5000; Jackson ImmunoResearch) for 1 h at room temperature. Bands were detected by Pierce ECL (Thermo Fisher) and quantified using Scion Image by analyzing pixel density. Protein levels were normalized to GAPDH that was used as the protein loading control.

### FRET tau seeding assay

Tau RD P301S FRET Biosensor (ATCC® CRL-3275™) was purchased from ATCC (Manassas, VA) and used for FRET tau seeding assay. Cells were plated at 24-well plates and transduced with either brain lysates or various tau fractions using Lipofectamine 2000 (Invitrogen) for three days, following the protocol previously described with slight modifications [15]. For FRET flow cytometry, cells were harvested and fixed with 2% paraformaldehyde for 10 min at room temperature. Fixed cells were subsequently resuspended in flow cytometer buffer (Hank’s Balanced Salt Solution buffer with 2% fetal bovine serum) and run on Attune NxT Flow Cytometer (Thermo Fisher) using 405 nm, 488 nm lasers, and 405/50 nm, 525/50 nm filters. Integrated FRET density was quantified using the average intensity of signals and the percentage of cells that are positive for FRET, based on the previous protocol [15]. For confocal microscopy analysis, cells grown on poly-D-lysine-coated coverslips were fixed with 4% paraformaldehyde for 10 min at room temperature. Cellular nuclei were counterstained with Hoechst 33258 (1 μg/ml, Life Technologies). Images were obtained on a Zeiss LSM 880 confocal microscope.

### Immunodepletion of tau

CNBr-activated Sepharose™ 4B agarose beads (GE Healthcare, Chicago, IL) were coupled with E1 tau antibody according to manufacturer’s protocol. Prior to use, antibody-coupled beads were equilibrated in PBS. Brain lysates were incubated with antibody-coupled beads overnight at 4 °C. Supernatant was collected as “tau-immunodepleted” sample and used for further analyses.

### Meso scale discovery (MSD) immunoassay

The level of total tau in samples was measured using the sandwich immunoassay with the Meso Scale Discovery System (Meso Scale Diagnostics, Rockville, Maryland). In brief, a 96-well MSD plate was coated with the capture antibody (E1) and incubated overnight. The following day, the plate was added with blocking buffer to reduce non-specific binding. Wells were washed and added with standards (recombinant tau) or samples for measurement. After incubation, wells were washed and added with the SULFO-TAG-labeled detection antibody (HT7; antibody was conjugated to the SULFO-TAG according to manufacturer’s protocol). MSD Read Buffer was added to wells to read tau levels using the MSD Sector Imager 2400. The light emission was read at 620 nm after electrochemical stimulation.

### Human brain tissues

All human postmortem brain tissues were obtained from the brain bank at Mayo Clinic Jacksonville. Frozen medial frontal cortex tissues were incubated in Hibernate A with collagenase to loosen up the tissue, and subsequently lysed as previously described [4]. Protein concentration was determined by BCA assay prior to their use for the FRET tau seeding assay or for immunoblotting.

### Immunohistochemistry

The human brain samples had been fixed in 10% formalin and embedded in paraffin wax. Tissues were sectioned (5 μm thickness) and mounted on glass slides. For immunostaining, tissue sections were first deparaffinized in xylene and rehydrated in a graded series of alcohols. For antigen retrieval, sections were steamed in citrate buffer (pH 6) for 30 min, and were subsequently incubated in 0.03% hydrogen peroxide to block endogenous peroxidase activity. Immunostaining of sections were performed using the DAKO Autostainer (DAKO North America, Carpinteria, CA) and the DAKO EnVision + HRP system, followed by dehydration step. The stained slides were then cover-slipped, and scanned with the Leica Aperio AT2 Slide Scanner (Leica Biosystems, Wetzlar, Germany). A color deconvolution algorithm from Aperio ImageScope software was used to analyze CP13 immunoreactivity indicative of p-tau burden.

### Isolation of sarkosyl-insoluble fraction from human brain tissues

Brain tissues (150 mg) were homogenized in Buffer A (10 mM Tris-HCl, 80 mM NaCl, 1 mM MgCl_2_, 1 mM EGTA, 0.1 mM EDTA, 100 mM DTT, 1 mM PMSF, protease inhibitor cocktail, and phosphatase inhibitor cocktails II and III) and ultracentrifuged at 150,000 x g for 70 min at 4 °C using the TLA-110 rotor. Supernatant (S1) was kept as “soluble fraction.” The pellet (P1) was resuspended in Buffer B (10 mM Tris-HCl, 850 mM NaCl, 1 mM EGTA, 10% sucrose) and centrifuged at 14,000 x g for 10 min at 4 °C to remove debris. The pellet (P2) was kept for potential future analyses, and the supernatant was collected to be incubated with 1% sarkosyl for 1 h at room temperature. After incubation, the sample was ultracentrifuged again at 150,000 x g for 40 min at 4 °C using the TLA-110 rotor. The supernatant (S2) was collected as “sarkosyl-soluble fraction”. The pellet (P3) was resuspended in ice-cold PBS and sonicated as the “sarkosyl-insoluble fraction.” Tau fractions were subject to tau MSD assay before being used in other experiments.

### Electron microscopy of tau filaments

To confirm the presence of tau filaments in sarkosyl-insoluble fraction using electron microscopy, samples (1:20 dilution of the original fraction) were absorbed onto a 400 mesh carbon/Formvar grid (Electron Microscopy Sciences, Hatfield, PA) for 30 s and stained with 2% uranyl acetate for 45 s. Images were obtained at high magnification using a Philips 208S electron microscope and a Gatan digital camera.

### Construct generation and AAV production

The K317 N mutant tau construct was generated from the wild-type tau-V5 parent construct using the QuikChange Mutagenesis kit (Agilent Technologies, Clara, CA), following the manufacturer’s protocol. The sequence was verified using ABI3730 with Big Dye chemistry, according to the manufacturer’s protocol (Applied Biosystems, Foster City, CA, USA). AAV was produced following our previous protocol [6]. In short, the TauK317 N expression plasmid was cloned into an AAV vector that includes the cytomegalovirus enhancer/chicken β-actin promoter, a woodchuck post-transcriptional regulatory element, and the bovine growth hormone polyA. HEK293T cells were transfected with AAV helper plasmids for 48 h, and the virus was isolated using a discontinuous iodixanol gradient. The genomic titer was determined by quantitative PCR.

### AAV transduction in primary mouse astrocytes

AAV was added to astrocytes at 500,000 MOI (multiplicity of infection) in reduced serum medium (DMEM + 2% FBS + 1% Pen/Strep) of half the usual volume for the particular surface area. Following 4 h of incubation at 4 °C, equal amount of fresh astrocyte growth medium (DMEM + 10% FBS + 1% Pen/Strep) was added to the culture. Media was changed with the fresh growth medium 48 h after the addition of AAV. To reach optimal AAV expression levels, astrocytes were cultured for 7 days total before experiments.

### Intracellular tau aggregation assay

For primary mouse astrocytes, cells were first incubated in starvation medium for 1 h and subsequently added with sarkosyl-insoluble fraction of AD or GGT (100 ng total tau based on MSD assay). After 4 h of incubation, cell media was changed to normal growth medium. After 48 h of incubation in total, cells were washed with ice-cold PBS and trypsinized to remove any residual tau from cell surface. Cells were subject to either confocal microscopy analysis or triton fractionation. For HEK293T cells, cells were first transfected with tau plasmid using Lipofectamine 2000 (Invitrogen) following manufacturer’s protocol. When serum-free media was changed to fresh growth medium 4 h after the addition of Lipofectamine/DNA cocktail, sarkosyl-insoluble (P3) fraction of AD or GGT (100 ng total tau based on MSD assay) was added to cells. Cells were harvested 48 h after the transfection for further analysis.

### Triton fractionation

Similar to the protocol described previously [32], cells were harvested and resuspended in the triton buffer A (50 mM Tris HCl [pH 7.4], 274 mM NaCl, 5 mM KCl, 5 mM EDTA, 1% Triton-X-100, 1 mM PMSF, protease inhibitor cocktail, and phosphatase inhibitor cocktails II and III). Samples were ultracentrifuged at 100,000 x g for 30 min at 4 °C and the supernatants were collected as “triton-soluble fraction.” The pellets were washed with 400 μl of triton buffer A to completely remove supernatant and were again ultracentrifuged at 100,000 x g for 30 min at 4 °C. The supernatants were completely removed and the pellets were resuspended in triton buffer B (buffer A with 1% final concentration of SDS) as “triton-insoluble fraction.” Both triton-soluble and insoluble fractions were subject to western blot for further analyses.

### Immunofluorescence staining and quantification of primary astrocytes with tau aggregates

Astrocytes grown on poly-D-lysine-coated coverslips were trypsinized to remove residual tau on cell surface and fixed with 4% paraformaldehyde for 10 min at room temperature. Cells were subsequently permeabilized with 0.5% Triton X-100/PBS for 10 min at room temperature. After blocking with non-fat dry milk in 0.2% Triton X-100/PBS for 1 h at room temperature, cells were incubated with primary antibody overnight at 4 °C and washed. Cells were then incubated with the corresponding Alexa Fluor 488- or 568-conjugated donkey anti-species secondary antibodies (1:1000, Molecular Probes, Eugene, OR) for 2 h at room temperature. Cellular nuclei were stained with Hoechst 33258 (1 μg/ml). Images were obtained using a Zeiss LSM 700 laser scanning confocal microscope. To quantify the percentage of astrocytes with E1-positive puncta, the number of GFAP-positive cells containing aggregated tau was counted in a blinded fashion from two independent experiments (60–100 cells were counted per experiment).

### Data analyses

Statistical analyses were performed using GraphPad Prism. For the FRET tau seeding assay with brain lysates, Kruskal-Wallis test was used followed by Dunn’s multiple comparison test. For the correlation between CP13 immunoreactivity and FRET signals induced by brain lysates, Spearman correlation was used. For the rest, differences among groups were analyzed using one-way ANOVA followed by Tukey’s multiple comparison test. The cutoff for statistical significance was *p* < 0.05.

## Results

### GGT brain lysates possess stronger seeding activity compared to other tauopathies

Given such striking differences in tau pathology between GGT and other tauopathies, we wanted to evaluate seeding competency of GGT brain lysates by using human postmortem brain tissue. Five GGT cases, including all three GGT subtypes among which is one case with a *MAPT* mutation (p.K317 N) [33], were selected for the analysis (Table 1). Neuropathological analysis using the CP13 antibody that detects tau phosphorylated on serine 202 was performed to determine GGT subtype classification based upon different anatomical and cellular distribution of GGIs across samples (Additional file 1: Figure S1). In addition to GGT cases, AD, PSP, and CBD cases as well as healthy controls were included in the analysis for comparison (Table 1). Total brain lysates prepared from frozen medial frontal cortex tissues were tested for tau seeding capacity using the fluorescence resonance energy transfer (FRET)-based tau biosensor cell line, a reporter cell line capable of detecting tau seeding activity in samples based on the induction of FRET signal as well as the formation of GFP-positive puncta [15].

**Table 1:** Information of samples used in the study

Sample	PathDx	Age at death	Sex	Braak NFT Stage	MAPT mutation	GGT subtype
Control 1	Normal	63	M	III		
Control 2	Normal	81	M	II		
AD 1	AD	68	M	VI		
AD 2	AD	72	F	V-VI		
AD 3	AD	81	F	VI		
AD 4	AD	91	F	VI		
PSP 1	PSP	59	M	II		
PSP 2	PSP	66	M	II		
CBD 1	CBD	67	M	II		
CBD 2	CBD	69	M	II		
GGT1	GGT	82	M	0-I		GGT subtype I
GGT2	GGT	55	F	V		GGT subtype III
GGT3	GGT	69	M	II		GGT subtype III
GGT4	GGT	68	F	III		GGT subtype II
GGT5	GGT	69	F	II-II	MAPT K317 N	GGT subtype III

**Fig. 1:**
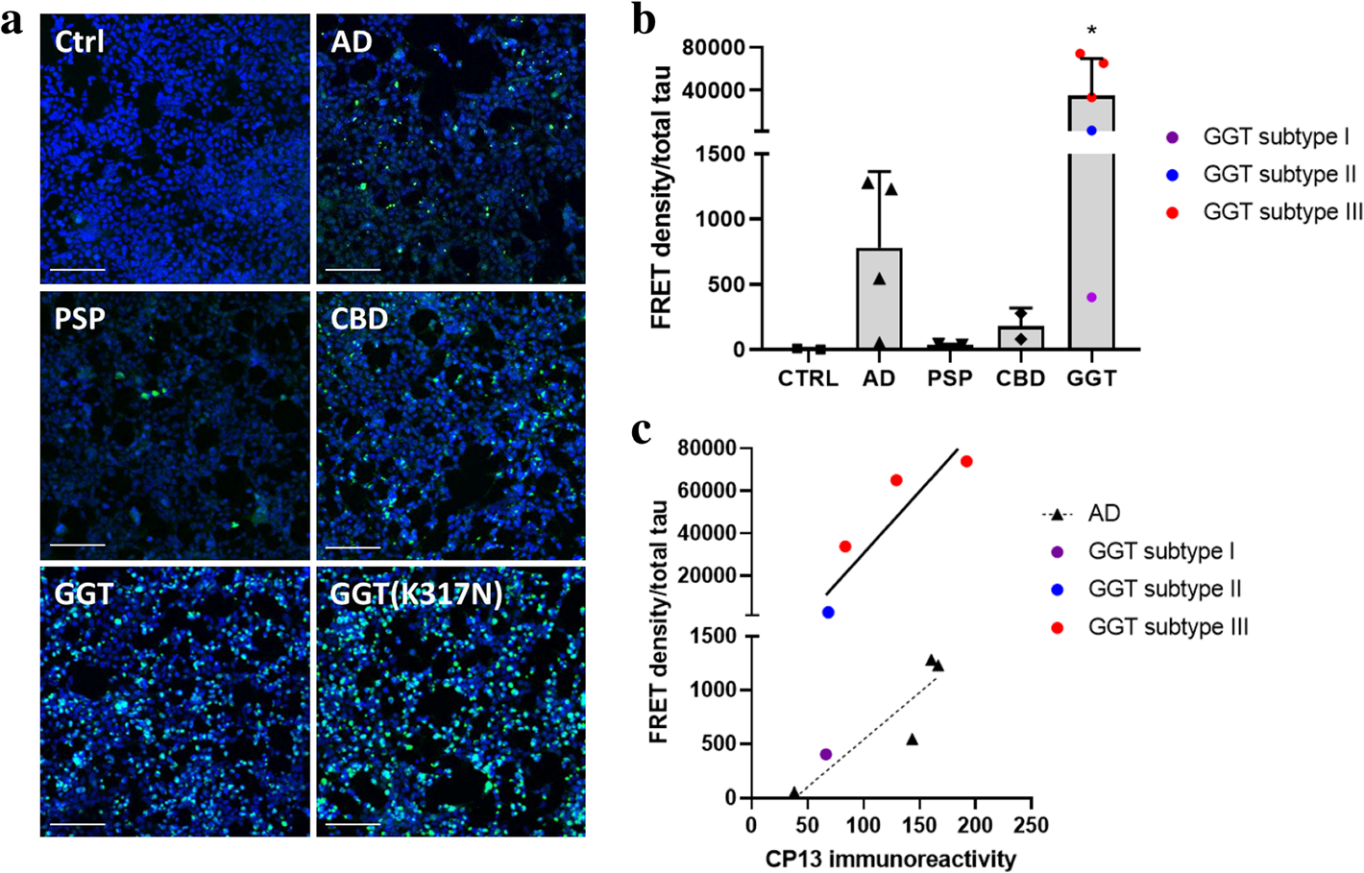
GGT brain lysates contain significantly higher tau seeding potency compared to other tauopathies. **a** Incubation of the tau biosensor cell line with brain lysates of sporadic or mutant (p.K317 N) GGT cases, resulted in induction of numerous GFP-positive puncta that are indicative of robust tau seeding. Representative confocal microscopy images are shown (scale bar = 100 μm). **b** Significant tau seeding activity of GGT brain lysates was measured by the FRET flow cytometry assay. FRET density was normalized to the amount of total tau present in the sample. Data are presented as mean ± SD (*p < 0.05 compared to control). **c** Analysis of p-tau burden from corresponding GGT and AD brain sections stained with CP13 p-tau antibody (pS202) demonstrated that GGT cases with similar p-tau burden to AD cases still induced robust FRET signals (R = 1, *p* = 0.0167 [GGT]; R = 0.8, p = ns [AD])

Surprisingly, cells incubated with GGT brain lysates showed significant induction of tau seeding activity by forming numerous GFP-positive puncta, a response that was considerably reduced or absent in cells incubated with brain lysates of other tauopathies or healthy controls (Fig. 1a). Similar results were obtained with the FRET flow cytometry assay, in which brain lysates from GGT cases induced very robust FRET signals (normalized to total tau amount in samples measured by Meso Scale Discovery immunoassay) that were significantly higher compared to other brain lysates, indicative of robust tau seeding competency of GGT samples (Fig. 1b). Of note, while the mean FRET signal induced by the GGT group was significantly higher than other tauopathies, individual FRET signals induced by GGT samples were heterogeneous (Fig. 1b). In particular, three of the highest FRET signals were induced by GGT cases with subtype III (Fig. 1b), which is characterized by predominantly globular astrocytic inclusions over oligodendroglial inclusions in both motor and frontal cortex [2]. In contrast, the lowest and the second lowest FRET signals were induced by GGT subtype I and II, respectively, (Fig. 1b), that have lower prevalence of globular tau inclusions in astrocytes compared to subtype III [2].

Despite the individual heterogeneity of tau seeding activity observed across GGT subtypes, the averaged tau seeding activity of GGT was much higher than other tauopathies. Specifically, the mean FRET signal in cells treated with GGT brain lysates was more than 50-fold higher than cells treated with AD brain lysates, which had the second highest seeding competency (Fig. 1b). To investigate the basis for these differences in seeding competency between GGT and AD brain samples, the corresponding brain sections from cases used in the FRET tau seeding assay were examined for their phosphorylated tau (p-tau) burden by staining with the CP13 antibody (Additional file 1: Figure S2a, b). Analysis of p-tau burden not only revealed a positive correlation between CP13 immunoreactivity and FRET signals within all cases (Fig. 1c), but also demonstrated that despite a similar level of p-tau burden, GGT cases had much higher tau seeding activity than AD cases as assessed by FRET (Fig. 1c). Taken together, these data show that GGT brain lysates exhibit more potent tau seeding activity compared to other tauopathies.

### Tau is the major factor in seeding competency of GGT brain lysates

**Fig. 2:**
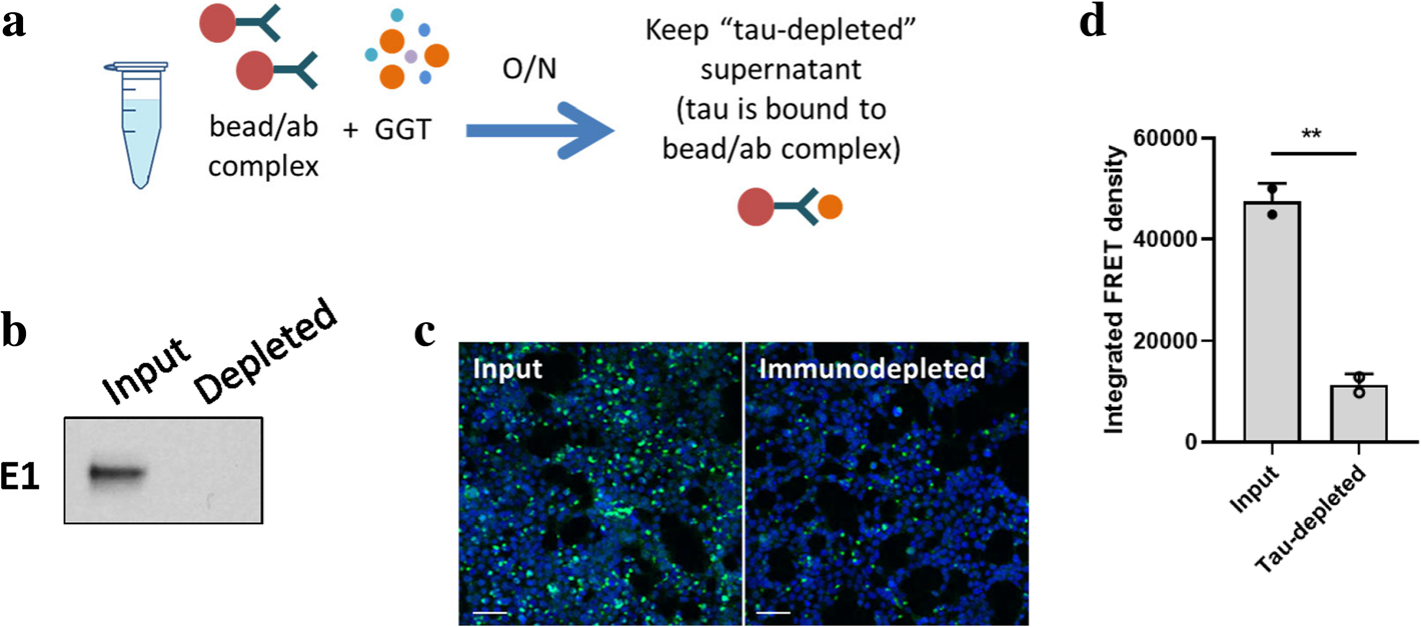
Tau is the main contributor of tau seeding competency of GGT brain lysates. **a** A schematic diagram depicting the process of immunodepletion of tau from GGT brain lysates. **b** Successful removal of tau from the sample confirmed by Western blot using a human tau-specific antibody E1 (19–33 amino acid). **c** Representative confocal images showing formation of much fewer seeding-induced puncta in the tau biosensor cell line upon incubation with tau-immunodepleted GGT brain lysates (scale bar = 100 μm). **d** A significant decrease in tau seeding activity following immunodepletion of tau from GGT brain lysates, as quantified by the FRET flow cytometry assay. Data are presented as mean ± SD (**p < 0.01)

Although the tau biosensor cell line responds specifically to tau seeds [15], we aimed to rule out the possibility that factors other than tau in GGT brain lysates might promote the observed seeding activities. A previous study confirmed antibody-mediated tau immunodepletion from samples was associated with a significant reduction in seeding activity in the biosensor cell line [22]. For this experiment, we used a polyclonal tau antibody to remove tau from GGT brain lysates to confirm altered tau seeding competency. Agarose beads coupled to a human tau-specific antibody (E1), which detects amino acid residues 19 to 33 of tau in a phosphorylation-independent manner, were incubated with the same GGT brain lysates that had induced the highest FRET signals (Fig. 2a). Following confirmation by Western blot that tau was successfully immunodepleted from the sample (Fig. 2b), tau-depleted GGT brain lysates were subsequently tested for tau seeding activity using the tau biosensor cell line. After immunodepletion, GGT brain lysates induced far fewer puncta as observed by confocal microscopy (Fig. 2c), and were also associated with an estimated 75% reduction in FRET signal compared to the original GGT brain lysates (Fig. 2d). While it can be speculated that some residual FRET signals were still being induced by tau species remaining in the sample that could not be immunodepleted with the E1 antibody due to unavailability of the epitope, these results collectively suggest that the robust tau seeding activity that characterizes GGT brain lysates is predominantly mediated by tau, rather than other factors in the sample.

### Aggregates induced by GGT brain lysates have a distinct morphology

**Fig. 3:**
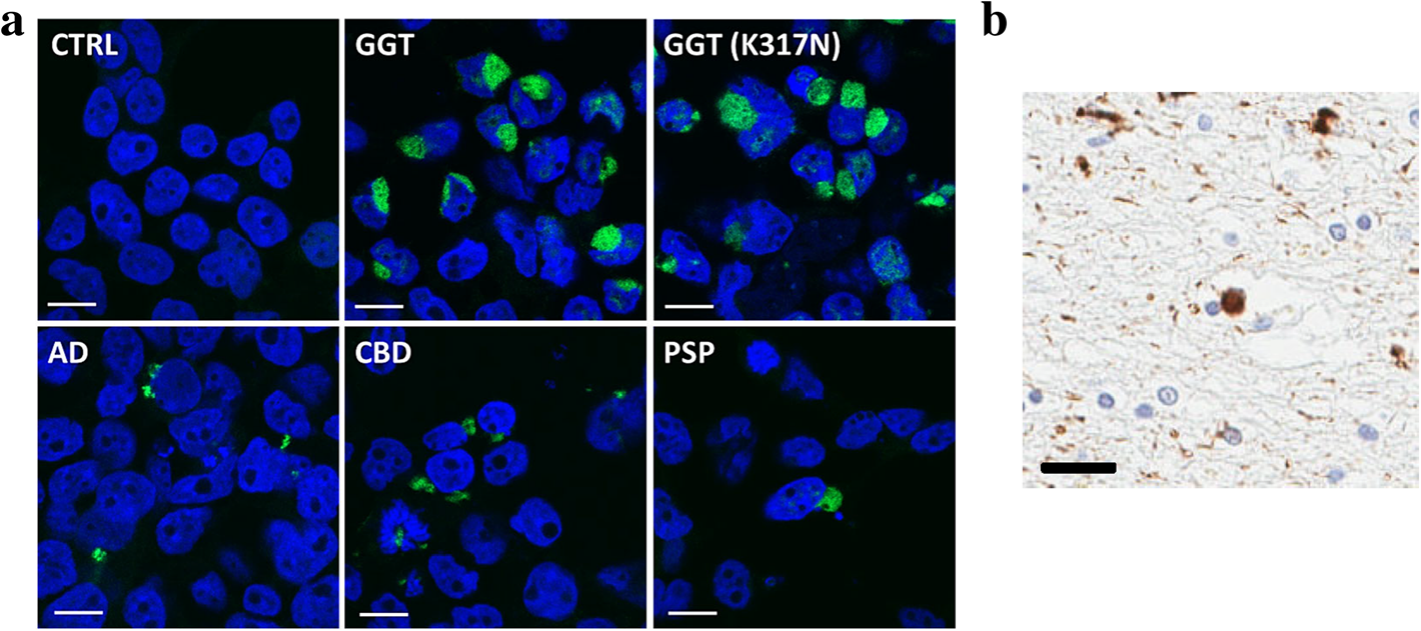
Tau aggregates induced by GGT brain lysates display distinct morphology. **a** A large, distinctively globular morphology of tau aggregates in the tau biosensor cell line induced by sporadic or mutant GGT brain lysates. Representative confocal images illustrate their markedly different morphology compared to those induced by AD, CBD, or PSP brain lysates (scale bar = 10 μm). **b** Tau-immunopositive, globular cytoplasmic inclusion in GGT brain sections stained with CP13 p-tau antibody (pS202) (scale bar = 10 μm)

Given that the tau biosensor cell line was previously described to recapitulate differences in putative tau “strains” that lead to various tauopathies [27], we compared the morphological characteristics of aggregates induced by brain lysates from different tauopathies as in original studies with this cell line. Among the tau inclusions formed in the tau biosensor cell line following incubation with brain lysates, aggregates induced by GGT brain lysates displayed a distinctive globular appearance compared to those induced by the other tauopathies (Fig. 3a). Of note, the morphology of the inclusions was reminiscent of globular inclusions observed in brain sections from GGT cases (Fig. 3b). In addition, aggregates in the tau biosensor cell line exposed to GGT brain lysates were larger than aggregates induced by other tauopathies (Fig. 3a). The distinct morphological features of GGT-induced tau aggregates suggest that unique tau species drive the characteristic tau pathology of GGT.

### Characterization of seeding properties of different tau species implicated in GGT

**Fig. 4:**
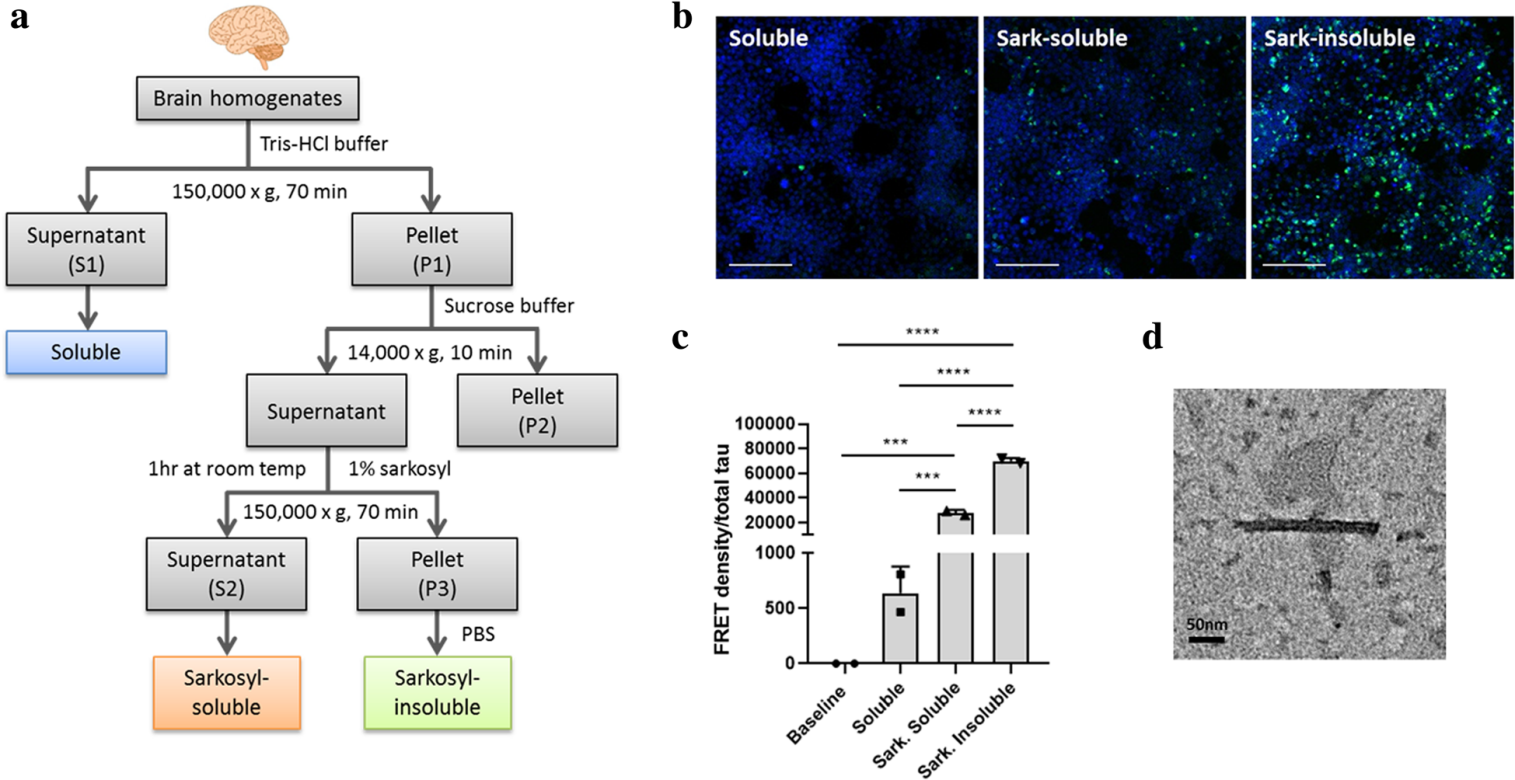
Sarkosyl-insoluble, fibril-like tau species implicated in GGT has the strongest seeding property**. a** A schematic diagram depicting the isolation method for different tau species from the brain samples. **b** Representative confocal images showing formation of the most seeding-induced puncta in the tau biosensor cell line upon incubation with sarkosyl-insoluble GGT-tau (scale bar = 100 μm). **c** The strongest tau seeding activity was detected in sarkosyl-insoluble GGT-tau as measured by the FRET flow cytometry assay. FRET density was normalized to the amount of total tau present in the sample. Data are presented as mean ± SD (***p < 0.001; ****p < 0.0001). **d** A representative electron microscopy (EM) image showing straight tau filaments in the sarkosyl-insoluble GGT-tau fraction

As brain lysates inevitably contain various types of tau species ranging from monomeric to multimeric tau, we aimed to characterize the seeding properties of different tau species implicated in tau pathology of GGT. From the GGT case with the highest FRET signal, soluble (S1), sarkosyl-soluble (S2), and sarkosyl-insoluble (P3) fractions were isolated, with each fraction representing monomeric, oligomeric, and fibrillar GGT-tau, respectively (Fig. 4a). These fractions with GGT-tau of different aggregation states were subsequently tested for their seeding competency in the tau biosensor cell line. Interestingly, the FRET tau seeding assay detected differences in FRET density across different tau species isolated from the same sample. P3 GGT-tau induced the most puncta in cells, followed by S2 GGT-tau and then by S1 GGT-tau (Fig. 4b). In accordance to puncta formation observed by confocal microscopy, FRET analysis revealed that P3 GGT-tau induced the highest FRET signal compared to other tau species (Fig. 4c). Electron microscopy confirmed that the P3 GGT-tau fraction contained fibrillar tau species (Fig. 4d), characterized by straight tau filaments typical of GGT [33].

### Insoluble GGT-tau can promote intracellular aggregation of mutant tau expressed in primary astrocytes

**Fig. 5:**
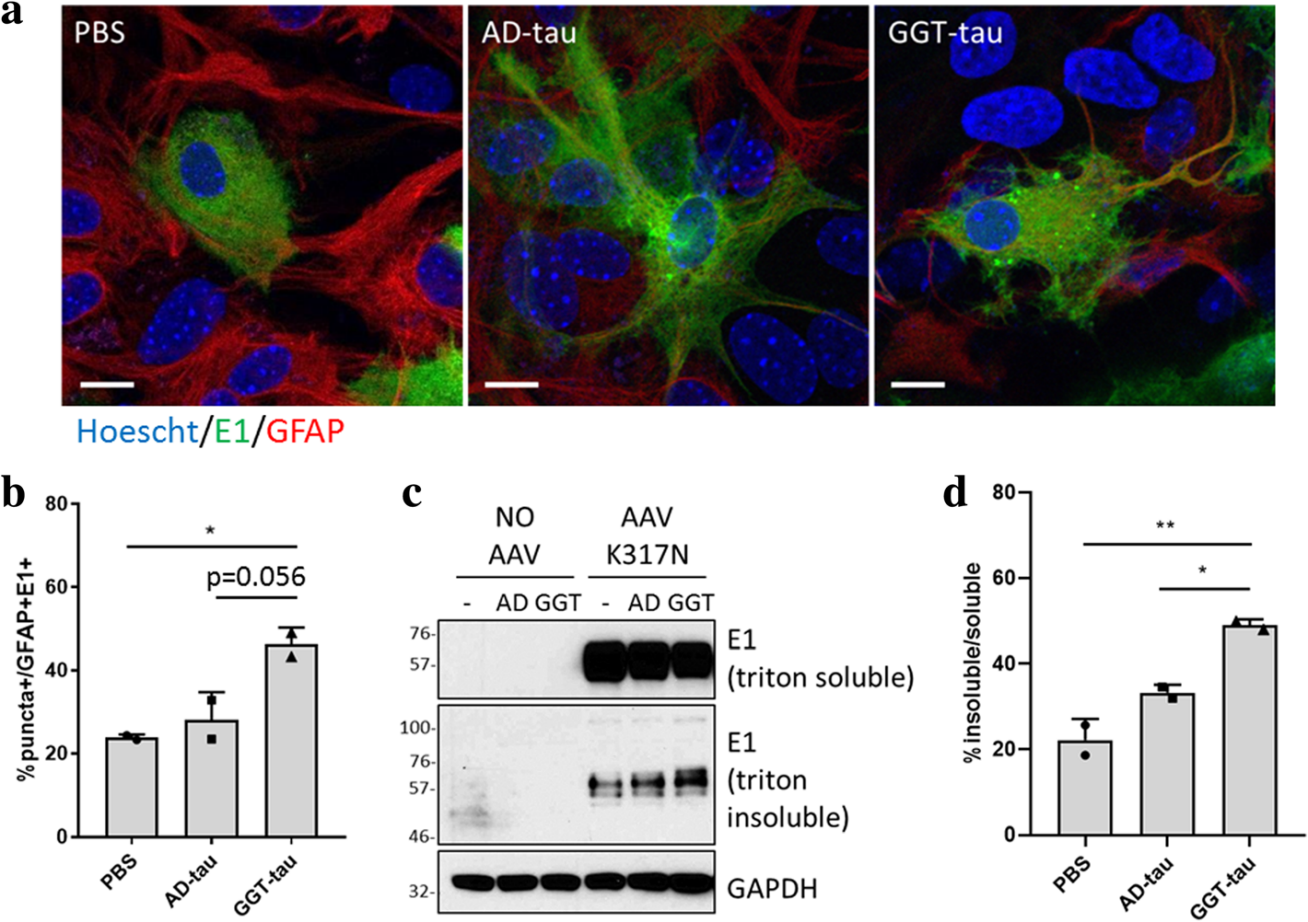
Sarkosyl-insoluble GGT-tau promotes intracellular tau aggregation in primary mouse astrocytes. **a** Representative confocal images showing primary mouse astrocytes transduced with AAV-Tau^K317N^ and subsequently treated with sarkosyl-insoluble GGT-tau or AD-tau for comparison. E1 staining for human tau is in green and GFAP staining for astrocytes is in red. Nuclei were stained with Hoechst (scale bar = 10 μm). **b** Quantification of percentage of astrocytes that are transduced with AAV-Tau^K317N^ and display tau seeding-like puncta (*p < 0.05). **c-d** Changes in the level of tau aggregation in AAV-transduced astrocytes upon treatment with either sarkosyl-insoluble GGT-tau or AD-tau were detected by Western blot (**c**) and quantified based on band intensity (*p < 0.05; **p < 0.01) (**d**)

Given the characteristic glial tau pathology implicated in GGT [2], primary mouse astrocyte cultures were selected to investigate tau seeding competency of GGT-tau. To better assimilate the GGT-like environment, we generated adeno-associated virus (serotype 9; AAV9) that expresses full-length tau with the mutation K317 N (c.951G > C), a *MAPT* mutation reported in GGT [33]. Primary mouse astrocytes were transduced with AAV9-Tau^K317N^ for 7 days to allow robust expression of Tau^K317N^. Then, cells were exposed to either P3 GGT-tau or P3 AD-tau, as well as PBS, for comparison (Additional file 1: Figure S3). Co-staining of cells with a human tau-specific antibody (E1) and the astrocytic marker GFAP confirmed that mouse astrocytes were effectively expressing human Tau^K317N^ (Fig. 5a). Importantly, incubation with P3 GGT-tau resulted in an approximately two-fold increase in the number of astrocytes with tau-positive puncta compared to the PBS group (Fig. 5a, b). In contrast, the number of tau-positive puncta was not significantly increased in astrocytes treated with P3 AD-tau (Fig. 5a, b), emphasizing the strong seeding potency of GGT-tau. We also confirmed the high seeding activity of P3 GGT-tau biochemically by evaluating triton-soluble versus triton-insoluble tau levels in primary mouse astrocytes treated with P3 GGT-tau or AD-tau. Consistent with our analysis using confocal microscopy (Fig. 5a,b), there was a significant increase in the ratio of triton-insoluble to soluble tau in primary mouse astrocytes transduced with AAV9-Tau^K317N^ and treated with P3 GGT-tau, suggesting biochemical evidence that GGT-tau induced aggregation of human Tau^K317N^ (Fig. 5c, d).

As an additional approach to evaluate seeding competency of P3 GGT-tau, we transfected HEK293T cells with either wild-type or K317 N mutant tau, and subsequently treated cells with P3 GGT-tau or AD-tau. We found that P3 GGT-tau promoted robust aggregation of K317 N mutant tau overexpressed in HEK293T cells, which was confirmed by isolation of triton-soluble or insoluble tau from these cells (Additional file 1: Figure S4). Remarkably, P3 GGT-tau also promoted aggregation of wild-type tau overexpressed in HEK293T cells, albeit less than K317 N mutant tau (Additional file 1: Figure S4). Similar to observations in primary mouse astrocytes, P3 AD-tau did not promote aggregation of either wild-type or K317 N mutant tau in HEK293T cells (Additional file 1: Figure S4). Collectively, these results indicate that GGT-tau has very robust seeding potential in cells.

## Discussion

In this study, we investigated the seeding properties of tau protein from GGT, which we found to be markedly different from tau in other tauopathies. Specifically, brain lysates of GGT cases, including one case with *MAPT* mutation, demonstrated potent seeding activity in a tau biosensor cell line compared to the other tauopathies studied – AD, PSP, and CBD – as well as healthy controls. This characteristically robust seeding competency observed in GGT samples was particularly linked to GGT subtype III. As GGT cases did not have overtly higher p-tau burden compared to AD cases, which exhibited the second highest tau seeding competency, this suggests that abnormal forms of tau in GGT brain lysates possess uniquely strong seeding activity. Moreover, tau aggregates formed in the tau biosensor cell line upon treatment with the GGT brain lysates were morphologically distinct from those induced by other tauopathy samples, exhibiting a globular form reminiscent of cytoplasmic glial tau inclusions observed in postmortem brain tissue from GGT. In addition to confirmation that tau is the main seeding factor in GGT brain samples, we identified that sarkosyl-insoluble, fibrillar GGT-tau had the strongest seeding activity among different tau species studied. The fibrillar GGT-tau further demonstrated robust seeding properties in primary mouse astrocytes transduced with human mutant tau.

Accumulating evidence from previous studies strongly supports the existence of different tau conformers with distinct structural and seeding properties, which could explain the heterogeneous clinical and pathologic features of tauopathies [8, 14, 21, 27]. While future studies evaluating pathological heterogeneity in other tauopathies are warranted, a major advancement in structural biology, cryo-electron microscopy (cryo-EM) technology, has offered significant insight into molecular differences among tauopathies. The initial cryo-EM-based investigation described the atomic structure of tau filaments from AD postmortem brain [12]. A subsequent study showed differences in tau filaments from Pick’s disease (PiD) [9]. These findings were particularly interesting given that tau filaments in AD include both 3R and 4R tau isoforms, while they include predominantly 3R tau in PiD. These studies collectively accentuate the remarkably heterogeneous structural properties that tau filaments adopt in a disease-specific fashion, which may be linked to the clinicopathological diversity observed across tauopathies. As such, additional cryo-EM studies on the structure of tau filaments from GGT, especially in comparison to other more common 4R tauopathies, such as PSP or CBD, may provide key insights into the structural pathophysiology of tau protein.

Regarding the structural properties of GGT-tau, the fact that the K317 residue of tau is mutated in several GGT cases (p.K317 N [33] and p.K317 M [37]) may be of particular relevance. Interestingly, according to the cryo-EM study on AD tau filaments, the K317 residue is located at the interface where two protofilaments interact with each other to form straight tau filaments [9]. It is therefore feasible that altering the charge at this particular residue (lysine to asparagine or methionine; positive to uncharged residue) may inhibit adoption of the AD tau filament structure, instead favoring the formation of GGT tau filament structure that has yet to be resolved. Of note, it has been previously described that straight tau filaments from the K317 N GGT case are morphologically similar to those from sporadic GGT cases [33], which might support the existence of a common GGT tau filament structure. Future studies are needed to determine if mutant and sporadic GGT tau filaments have comparable atomic structures and biochemical properties, which could help elucidate the molecular basis of distinct pathological features of GGT.

Valuable lessons also come from studies on synucleinopathy, another neurodegenerative proteinopathy that is characterized by abnormal aggregation of α-synuclein. Similar to different cell-type specific presentations of tau inclusions in tauopathies, pathological α-synuclein can also display distinct neuronal versus glial preferences by aggregating in the form of neuronal Lewy bodies (LB) in Parkinson’s disease and dementia with Lewy bodies, or glial cytoplasmic inclusions (GCIs) in multiple system atrophy (MSA), respectively [10]. Recent studies on different biochemical and pathogenic properties between LB-like and GCI-like α-synuclein found that higher pathogenicity and stronger seeding properties were associated with GCI-like α-synuclein [23, 35]. These findings are highly relevant to our current study due to overlapping similarities between α-synuclein-positive GCIs in MSA and tau-positive GGIs in GGT in their pathological presentation, including morphology of aggregates and cell-type specificity [26]. Given that our findings on unique seeding properties of GGT-tau parallel studies on α-synuclein, a new area of investigation is to evaluate whether the cellular milieu in glial cells acts to drive formation of specific tau conformers that are highly seeding-potent and GGI-inducing, as demonstrated for GCI-like α-synuclein [23].

Although neurons express the highest levels of tau, tau is also expressed in astrocytes and oligodendrocytes at low levels, and is crucial for specific physiological functions [20]. In addition to GGI in GGT, glial tau abnormalities are also observed in other tauopathies, such as tufted astrocytes in PSP and astrocytic plaques in CBD, as well as coiled bodies in oligodendrocytes observed in several tauopathies [11, 36]. It is currently unknown whether glial tau inclusions are derived directly and solely from endogenous tau in astrocytes and oligodendrocytes or from uptake of extracellular neuronal tau. For instance, it has been shown that amyloid-beta, engulfed by astrocytes, can accumulate to form large inclusions due to inefficient digestion [31]. Similarly, it is possible that GGIs are the remnant of pathological neuronal tau taken up by glia and incompletely digested. Future studies investigating degradation of GGT-tau versus tau in other tauopathies may better elucidate the nature of GGIs and the distinct seeding properties of GGT-tau. One strategy to investigate this question is to identify unique interactors or binders to GGT-tau that do not bind AD-tau, which could be linked to impaired degradation of GGT-tau and subsequent formation of GGIs. Although we demonstrated with immunodepletion that GGT-tau is the major factor exerting seeding activity from GGT brain lysates, additional studies are needed to exclude the possibility that factors other than GGT-tau at least partially promote or influence the observed robust tau seeding competency through binding or interacting with GGT-tau. These factors could also stimulate changes in tau that favor a GGT-like conformation, independent of the aforementioned mutations at residue K317 of tau. Therefore, utilization of different quantitative proteomics approaches [16, 25, 30] to determine key interactors and binders (or lack thereof) for GGT-tau will provide new insight and may be essential to understand pathomechanisms underlying tau deposition in GGT.

## Conclusions

In conclusion, our findings demonstrate that GGT is characterized by tau with high seeding potency that could be linked to its distinct morphologic phenotype. In particular, pathological tau species from GGT have stronger seeding competency compared to other tauopathies, which is similar to enhanced seeding potency of GCI-type α-synuclein pathology in MSA demonstrated by recent reports [23, 35]. Our study is the first to investigate seeding properties of tau in GGT, providing novel insight into the heterogeneous nature of tau pathology in tauopathies. As GGT has only recently been classified as a distinct disease entity [2], several GGT cases may have been overlooked or classified as an alternate tauopathy. Future investigations into disease-relevant properties of GGT-tau, including potential structural differences between GGT-tau and other tauopathies, in addition to the identification of key interactors or binders specific for GGT-tau, will advance our understanding of tauopathies with pronounced glial tau pathology.

## Additional file

Additional file 1: **Figure S1.** Heterogeneous distribution of globular glial tau pathology across GGT samples. **Figure S2.** Phospho-tau staining of GGT and AD brain sections. **Figure S3.** Sarkosyl-insoluble AD-tau is fibril-like. **Figure S4.** Sarkosyl-insoluble GGT-tau promotes intracellular tau aggregation in HEK293T cells. (DOCX 2331 kb)

## Abbreviations

AAV: Adeno-associated virus
AD: Alzheimer’s disease
CBD: Corticobasal degeneration
cryo-EM: Cryo-electron microscopy
EM: Electron microscopy
FRET: Fluorescence resonance energy transfer
GCI: Glial cytoplasmic inclusions
GFP: Green fluorescent protein
GGI: Globular glial inclusion
GGT: Globular glial tauopathy
LB: Lewy bodies
MAPT: Microtubule associated protein tau
MSA: Multiple system atrophy
MSD: Meso Scale Discovery
PiD: Pick’s disease
PSP: Progressive supranuclear palsy
