# Supplementary material for: Tau exhibits unique seeding properties in globular glial tauopathy
Source: Acta Neuropathol Commun. 2019 Mar 7;7:36. doi: 10.1186/s40478-019-0691-9 (PMC6404306; doi:10.1186/s40478-019-0691-9)
Supplement: Supplementary file 1 — Figure S1. Heterogeneous distribution of globular glial tau pathology across GGT samples. Figure S2. Phospho-tau staining of GGT and AD brain sections. Figure S3. Sarkosyl-insoluble AD-tau is fibril-like. Figure S4. Sarkosyl-insoluble GGT-tau promotes intracellular tau aggregation in HEK293T cells. (DOCX 2331 kb) [file 40478_2019_691_MOESM1_ESM.docx]

**Supplementary figures**

**Fig. S1 Heterogeneous distribution of globular glial tau pathology across GGT samples.**

**Fig. S2 Phospho-tau staining of GGT and AD brain sections.**

**Fig. S3 Sarkosyl-insoluble AD-tau is fibril-like.**

**Fig. S4 Sarkosyl-insoluble GGT-tau promotes intracellular tau aggregation in HEK293T cells.**

**Figure S1**


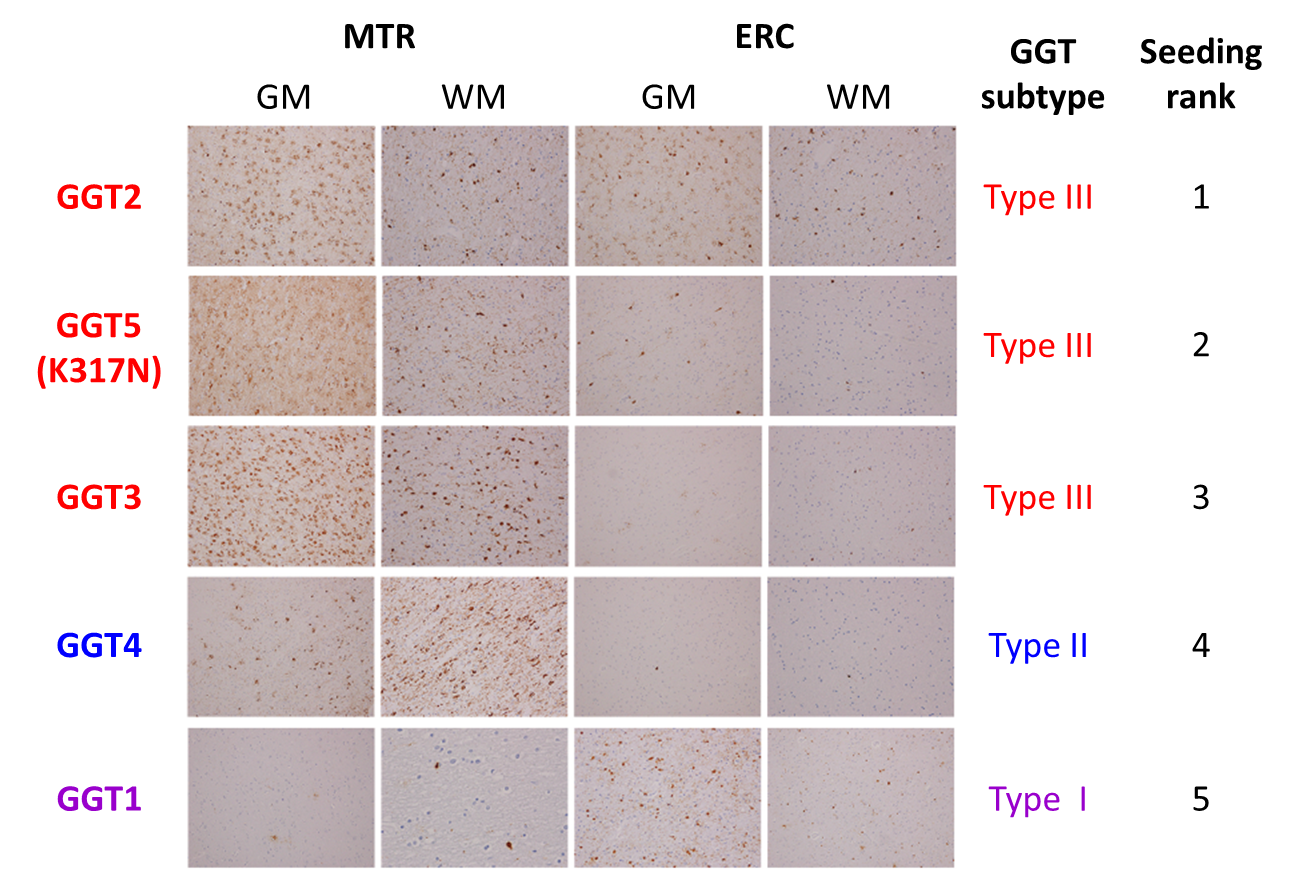


**Fig. S1 Heterogeneous distribution of globular glial tau pathology across GGT samples.** Motor cortex and entorhinal cortex of GGT cases used in the FRET tau seeding assay were stained with CP13 p-tau antibody to examine different distribution of tau pathology between motor and entorhinal cortex, as well as grey and white matter (MTR motor cortex; ERC entorhinal cortex; GM grey matter; WM white matter). Immunohistochemical images of GGT cases are shown in the order of tau seeding activity rank measured by the FRET tau seeding assay (1 – highest; 5 – lowest).

**Figure S2**

**
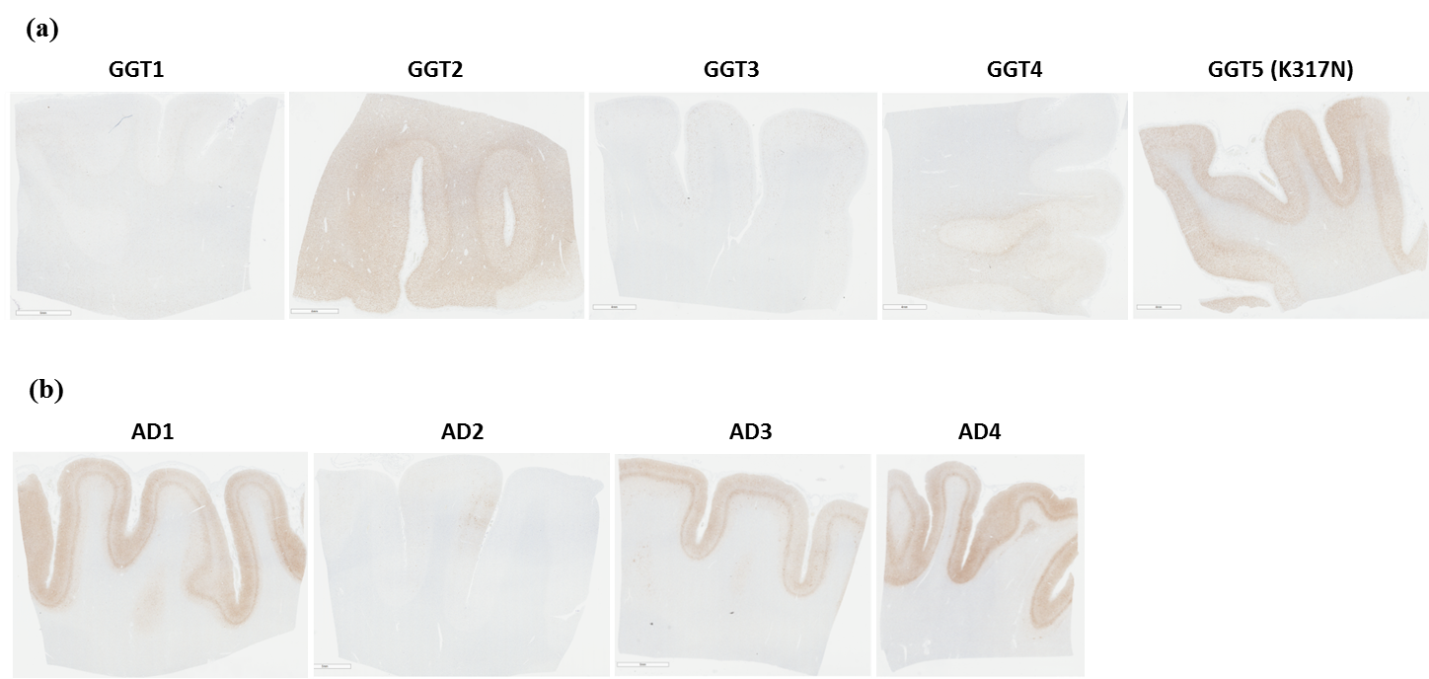
**

**Fig. S2 Phospho-tau staining of GGT and AD brain sections.** Brain sections of GGT cases (a) and AD cases (b) that correspond to the samples used in the FRET tau seeding assay were stained with CP13 p-tau antibody to determine p-tau burden in each case.

**Fig. S3**


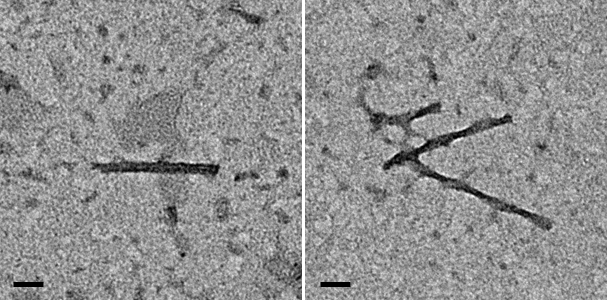


**Fig. S3 Sarkosyl-insoluble AD-tau is fibril-like.** A representative EM image confirming the presence of tau filament in the sarkosyl-insoluble AD-tau fraction used in the experiment. The filaments have a paired helical filament morphology, which is markedly different from straight filaments in GGT (scale bar = 50 nm).

**Fig. S4**


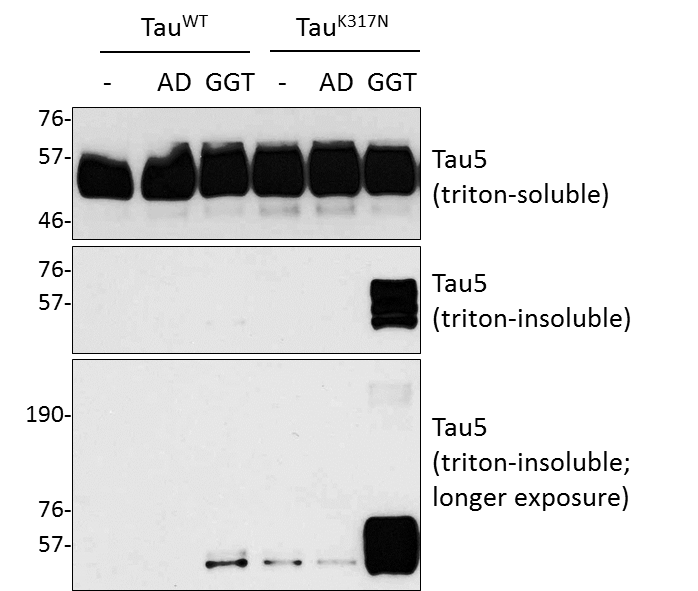


**Fig. S4 Sarkosyl-insoluble GGT-tau promotes intracellular tau aggregation in HEK293T cells.** Seeding potency of sarkosyl-insoluble (P3) GGT-tau was demonstrated in HEK293T cells overexpressing wild-type or K317N mutant tau. Western blot using total tau antibody tau5 (210-241 amino acid) detected a robust increase in triton-insoluble tau in cells treated with P3 GGT-tau, compared to cells treated with P3 AD-tau or PBS.
